# Adherence, tolerance and effectiveness of two different pelvic support belts as a treatment for pregnancy-related symphyseal pain - a pilot randomized trial

**DOI:** 10.1186/s12884-015-0468-5

**Published:** 2015-02-15

**Authors:** Natasha AMS Flack, E Jean C Hay-Smith, Mark D Stringer, Andrew R Gray, Stephanie J Woodley

**Affiliations:** Department of Anatomy, University of Otago, Dunedin, New Zealand; Department of Medicine, University of Otago, Wellington, New Zealand; Department of Women’s and Children’s Health, University of Otago, Dunedin, New Zealand; Department of Preventive and Social Medicine, University of Otago, Dunedin, New Zealand

**Keywords:** Pubic symphysis pain, Pelvic support belts, Pain, Function, Disability, Tolerance, Effectiveness, Adherence

## Abstract

**Background:**

Pregnancy-related pubic symphysis pain is relatively common and can significantly interfere with daily activities. Physiotherapist-prescribed pelvic support belts are a treatment option, but little evidence exists to support their use. This pilot compared two pelvic belts to determine effectiveness (symptomatic relief), tolerance (comfort) and adherence (frequency, duration of use).

**Methods:**

Unblinded, 2-arm, single-center, randomized (1:1) parallel-group trial. Twenty pregnant women recruited from the community (Dunedin, New Zealand), with physiotherapist-diagnosed symphyseal pain, were randomly allocated to wear either a flexible or rigid belt for three weeks. One author, not involved in data collection, randomized the allocation to trial group. The unblinded primary outcome was the Patient Specific Functional Scale (PSFS). Secondary outcomes were pain intensity during the preceding 24 hours and preceding week (visual analogue scale [VAS]), and disability (Modified Oswestry Disability Questionnaire [MODQ]). Duration of use (hours) was recorded daily by text messaging. Participants were assessed at baseline, by weekly phone interviews and at intervention completion (three weeks). To assess comfort, women wore the alternate belt in the fourth week.

**Results:**

Twenty pregnant women (mean ± SD age, 29.4 ± 6.5 years; mean gestation at baseline, 30.8 ± 5.2 weeks) were randomized to treatment groups (flexible = 10, rigid =10) and all were included in analysis. When adjusted for baseline, PSFS scores were not significantly different between groups at follow up (mean difference −0.1; 95% CI: −2.5 to 2.3; *p* =0.94). Pain in the preceding 24 hours reached statistical significance in favor of the flexible belt (VAS, p = 0.049). Combining both groups’ data, function and pain were significantly improved at three weeks (mean difference −2.3; 95% CI: 1.2 to 3.5; *p*< 0.001). Belts were worn for an average of 4.9 ± 2.9 hours per day; women preferred the flexible belt. No adverse events were reported.

**Conclusion:**

These preliminary results suggest the flexible pelvic support belt may be more effective in reducing pain and is potentially better tolerated than a rigid belt. Based on these data, a larger trial is both feasible and clinically useful.

**Trial registration:**

Australian New Zealand Clinical Trials Registry (ANZCTR) ACTRN12614000898651, 25th August, 2014.

**Electronic supplementary material:**

The online version of this article (doi:10.1186/s12884-015-0468-5) contains supplementary material, which is available to authorized users.

## Background

During pregnancy women may experience a variety of musculoskeletal complaints including low back pain [1-6] and/or pelvic girdle pain [1,7-13]. Pubic symphysis dysfunction is a distinct subgroup of pelvic girdle pain [1] and during pregnancy causes pain and limits everyday activities [14] in at least 3-8% of women [1,15]. Symptoms can be very debilitating, do not necessarily resolve after childbirth [1,7,15] and often recur in subsequent pregnancies [15,16]. Despite an adverse impact on quality of life [14,15,17], the cause of pregnancy-related symphyseal pain is poorly understood [18], with hormonal, genetic and/or biomechanical factors implicated in the pathogenesis [19] of this relatively common but under-estimated problem [15].

A theoretical model of pelvic function based on biomechanical and clinical research, highlights the interaction of the bony interlocking mechanisms of the joints of the pelvis and the support afforded by surrounding ligaments, fascia and muscles [20]. Insufficiencies in any of these components could result in abnormal pelvic motion [20,21], thereby generating pain [2]. Pelvic support belts have been used by physiotherapists to treat symphyseal pain during pregnancy and the postpartum period [14,22]. It is hypothesized that belts limit excessive motion by exerting an external force which compresses and stabilizes the joint(s) [2,23], potentially generating a self-bracing effect [21].

There is little empirical evidence to support the efficacy or effectiveness of pelvic support belts in the management of pregnancy-related symphyseal pain. In clinical practice, pelvic belts are typically used in conjunction with other treatments such as exercise or acupuncture [8-10,14,24], meaning their individual contribution is unknown. In addition, a variety of belts are available for use [8,9,14,23,25-27], yet it is not certain what type is most beneficial in terms of symptom relief and tolerance. To date, only one study has investigated the effects of a pelvic belt as a primary intervention in pregnant women [3] and one has reported their use in the treatment of symphyseal dysfunction [14]. However, most intervention studies have focused on pregnant women with generalized pelvic girdle pain [3,8,10,24,26]. In order to better understand the effects of a pelvic belt on pain and function, this pilot, unblinded (participants and researchers), 2-arm, single-center, randomized (1:1), parallel-group trial compared two pelvic support belts to determine effectiveness (symptomatic relief), tolerance (comfort) and adherence (frequency and duration of use) in women with pregnancy-related symphyseal pain.

## Methods

### Trial design

This pilot study was an unblinded (participants and researchers), single-center, 2-arm, randomized (1:1), parallel-group study conducted in Dunedin, New Zealand. Ethical approval was granted by the New Zealand Upper South A Regional Ethics Committee (reference URA/11/05/012) and registered in the Australian New Zealand Clinical Trials Registry (#ACTRN12614000898651), registered 25th August, 2014.

### Participants

**Figure 1:**
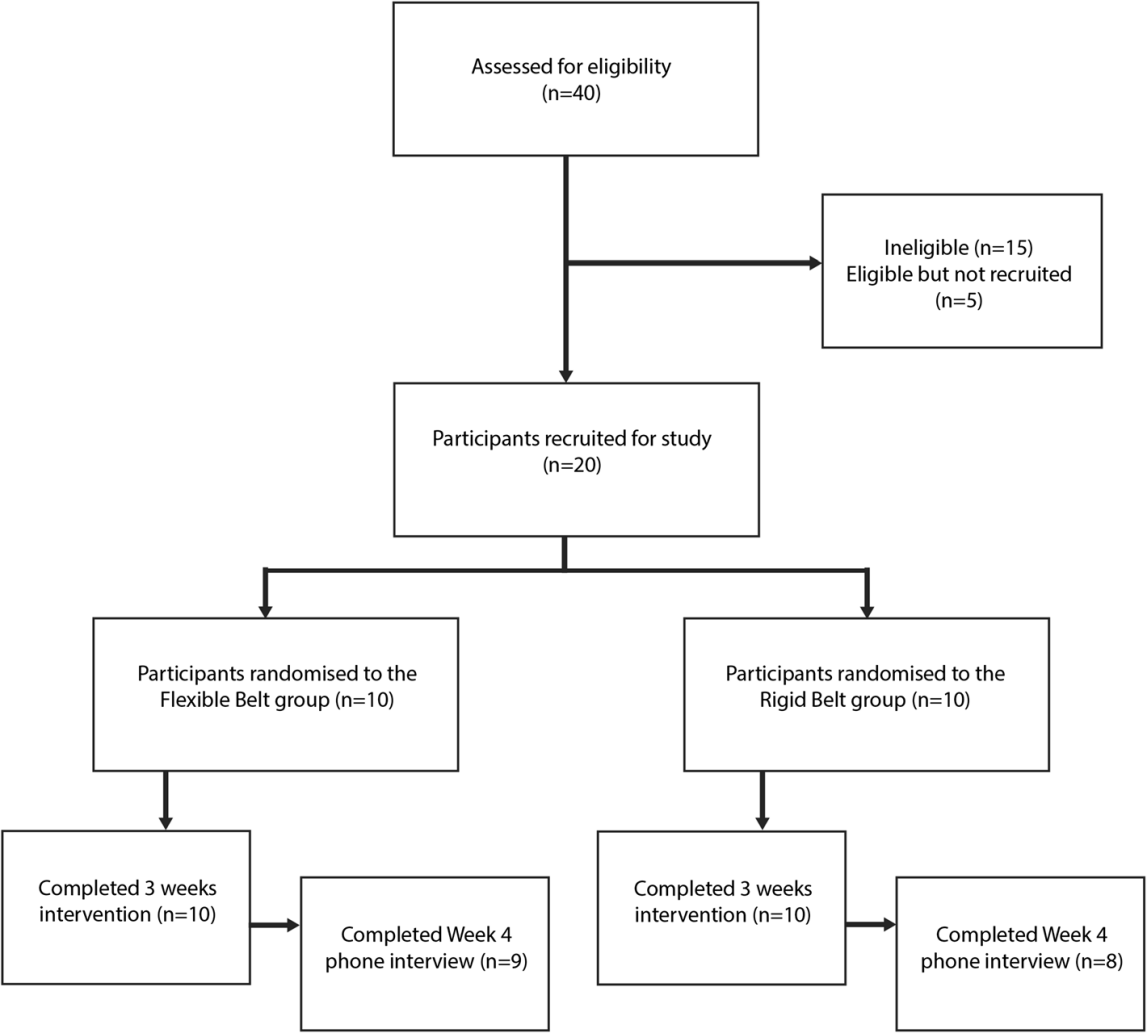
**Flow diagram showing the study recruitment process.**

Recruitment took place within the community in Dunedin, New Zealand by referral from Leading Maternity Carers (midwives) and advertisements placed in a local newspaper. Women were eligible to take part in the study if they were pregnant, at least 18 years of age, had experienced pubic symphyseal pain for at least two weeks (which was worse than any concurrent posterior pelvic pain), and had a positive response to at least two of three clinical tests: reproduction of pain from palpation [11], modified Trendelenburg’s test [11], active straight leg raise test [12,14,25] (as described in Additional file 1). Women with a known high-risk pregnancy, a history of major systemic bone disease and/or back or pelvic injury, a medical condition which contraindicated the use of a pelvic support belt (e.g. certain types of placenta previa), or those currently taking steroid medication were excluded. Figure 1 shows a flow diagram of the recruitment process.

### Assessment

After obtaining informed, written consent, participants completed a standardized baseline questionnaire (Additional file 2) which included pain history, a 10 cm Visual Analogue Scale (VAS) to quantify pain [28] intensity over the preceding 24 hours and the preceding week, and the Modified Oswestry Disability Questionnaire (MODQ) to determine the influence of symphyseal pain on activities of daily living [29,30]. The Patient Specific Functional Scale (PSFS) [31] was then completed in conjunction with the assessing physiotherapist (SW). Participants were tested for joint hypermobility using the nine-point Beighton Hypermobility Score (adapted from [32]) in which hypermobility was defined as a score of ≥4 points [33].

### Randomization

Using a computer-generated random number table and block sizes of 4 (produced by MS and concealed in sealed opaque envelopes, sequentially numbered), participants were assigned in a 1:1 allocation to either flexible or rigid belt. Following assessment and enrolment into the study, NF drew and opened the next sequentially numbered envelope and then communicated with SW about which belt each participant would be using.

**Figure 2:**
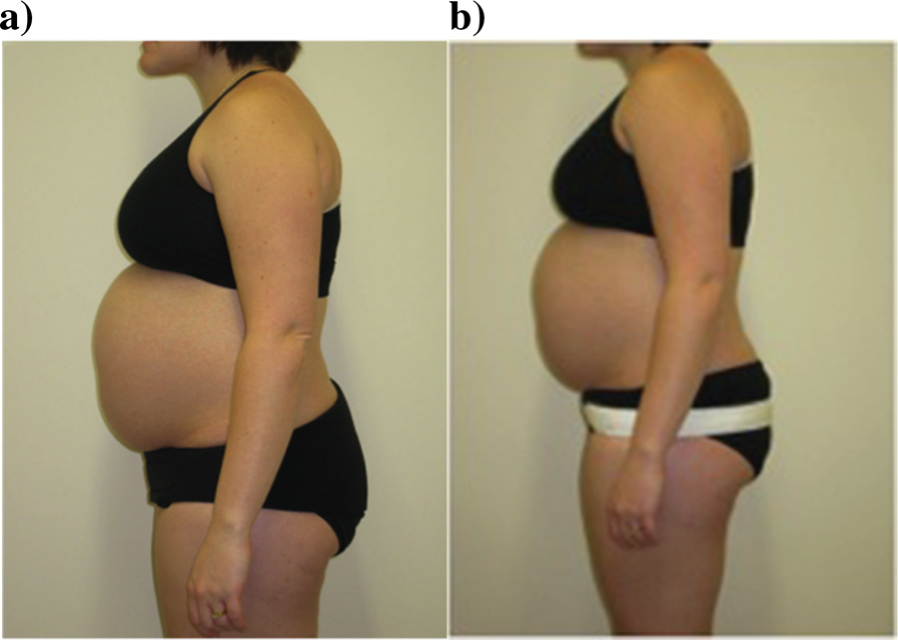
**The two types of pelvic support belts trialed in this study. (a)** Wider, more flexible “Smiley” belt made of neoprene material (Smiley Belt, www.smileybelt.co.nz, Havelock North, New Zealand. **(b)** Thinner, more rigid belt made of nylon webbing and lined with foam (LC symphysis pubis belt, The Orthotic Center New Zealand Limited, Greenlane, Auckland, New Zealand). Both belts were worn in the “low” position [34] over the level of the pubic symphysis. Specific consent was given by the woman pictured, for these photographs to be published in this article.

Intervention: Women were randomized to three weeks wear of either a wide, flexible neoprene support belt (Smiley Belt, www.smileybelt.co.nz, Havelock North, New Zealand; NZD $58.00) or a thinner, more rigid belt made of nylon webbing with foam lining (LC symphysis pubis belt, The Orthotic Center New Zealand Limited, Greenlane, Auckland, New Zealand; NZD $21.30). Participants were shown by a physiotherapist (SW) how to wear the belt, aligned over the pubic symphysis (the so-called ‘low position’ [34], Figure 2) and were advised to wear it whenever possible during waking hours.

Women were sent automated standardized daily text messages and asked to respond on the number of hours the belt had been worn, whether pain had decreased (yes/no/sometimes) and if functional activities were easier to perform (yes/no/sometimes). Responses to daily text messages were recorded. Weekly phone interviews were conducted (NF) to complete the PSFS and determine adherence (frequency and duration of use) and tolerance (comfort) of the belt.

### Follow up

At the conclusion of the intervention (three weeks), the un-blinded researcher (NF) met with participants to reassess the PFSF, and each woman self-completed the MODQ and VAS. Participants were then fitted with the alternate belt to wear for one week. Following this (week 4), a final phone interview was conducted which included the same questions as weeks 1 and 2, respectively, and participants were asked about belt preference (the proforma for the phone interviews is shown in Additional file 3).

### Outcomes

The primary outcome measure was the PSFS and the primary endpoint was three weeks. The PSFS has been shown to be a reliable and valid test for assessing disability and change in disability, and a 2-point difference in scores may be considered a minimal clinically important change [31]. For each group, mean scores for all outcome measures were calculated at baseline and at the end of week 3. Data were then transformed as described by Westaway et al. [31]. Secondary outcome measures were pain intensity rated on the 10 cm VAS for worst pain over the preceding 24 hours and the preceding week, and functional status determined by the MODQ score, interpreted using the specified guidelines [35].

### Sample size

Before a multi-center trial of pelvic support belts can be launched, it is essential to gather data on the most appropriate type of belt, symptomatic effectiveness, comfort, and adherence as well as to provide estimates of anticipated variability and correlations between repeated measures. Having 10 participants in each arm of this study was considered sufficient to obtain preliminary data to help with estimating power for a larger subsequent study.

### Statistical analysis

Data were entered into a spreadsheet. Appropriate descriptive statistics were derived for all variables. Linear regression models were used to examine differences in follow-up values between the two groups (flexible and rigid belts) after adjusting for baseline values. Model assumptions were assessed using histograms of residuals, plots of residuals against fitted values, and Levene’s test for equality of variance between groups. Where model residuals were positively skewed or demonstrated heteroscedasticity, natural logarithmic transformations were investigated, after adding one in the case of measures that included zero values. Overall changes (across both groups) in measures were examined using paired t-tests where there was no evidence of differences in change between groups and regression models using follow-up values while controlling for baseline values were used to assess any association with compliance. Stata 13.1 was used for all statistical analysis and all tests were performed using two-sided p < 0.05 as indicating statistical significance.

## Results

Recruitment took place in Dunedin, New Zealand from April 2011 to May 2012. Twenty participants were recruited, 10 of whom were randomized to wearing the flexible belt and 10 the rigid belt. All 20 participants were followed up at three weeks, with one participant not providing usable pain VAS scores in each group at follow-up. Three did not complete the phone interview in Week 4; two women had given birth and did not continue with the study and one woman was unable to be contacted. Baseline demographic data for the two belt groups and combined data for all participants are shown in Table 1 showing that the groups were comparable. No participants tested positive for joint hypermobility.

**Table 1:** **Mean (SD) baseline demographic data for all participants**

**Characteristic**	**Flexible belt group**	**Rigid belt group**	**All participants**
	**(n = 10)**	**(n = 10)**	**(n = 20)**
Mean age (years)	28.6 (5.6)	30.2 (7.6)	29.4 (6.5)
Mean BMI (kg/m^2^)	24.8 (3.8)	24.8 (4.2)	24.8 (3.9)
Mean gestation at baseline (weeks)	32.0 (4.8)	29.6 (5.5)	30.8 (5.2)
Mean gestational age at onset of symptoms (weeks)	24.4 (7.5)	22.7 (6.5)	23.6 (6.9)

*Abbreviations*: *SD* standard deviation.

Patient Specific Functional Scale scores (primary outcome) were not significantly different between the groups at follow up when adjusting for baseline (mean difference for flexible belt compared to rigid belt: −0.1; 95% CI: −2.5 to 2.3; *p* = 0.94). However, pain VAS scores (preceding 24 hours) decreased in the flexible belt group compared to the rigid belt group (ratio of geometric means 0.6, 95% CI: 0.4 to 1.0, p = 0.049). Scores for the other two measures (VAS preceding week, MODQ) were not significantly different between the groups (both p ≥ 0.454) (Table 2).

**Table 2:** **Mean (SD) outcome values for study groups at baseline and after 3 weeks intervention**

**Variable**	**Flexible belt**		**Rigid belt**		**Changes**		
	**Baseline**	**Follow-up**	**Baseline**	**Follow-up**	**Difference** ^**†**^	**95% CI**	**p-value**
PSFS	6.5 (1.6)	4.2 (2.9)	7.1 (1.6)	4.7 (2.4)	−0.1	−2.5, 2.3	0.938
Pain VAS (preceding 24 hours)	55.5 (24.0)	37.9 (26.6)	58.2 (24.4)	58.9 (22.3)	0.6^*^	0.4, 1.0	0.049
Pain VAS (preceding week)	68.7 (21.3)	53.0 (30.0)	71.6 (18.0)	64 (23.1)	0.8^*^	0.5, 1.4	0.478
MODQ	30.5 (15.7)	25.5 (19.6)	28.0 (14.7)	27.1 (15.5)	−3.9	−14.8, 6.9	0.454
Compliance (number of hours worn)	5.0 (2.1)		4.9 (3.6)		Combined (Flexible and Rigid)		4.9 (2.9)

*Log-transformed prior to statistical modelling due to skew and heteroscedasticity in residuals so difference is a ratio of geometric means with values < 1 indicating lower values in the flexible belt group.

Significance taken as *P* <0.05. *Abbreviations*: MODQ, Modified Oswestry Disability Questionnaire; PSFS, Patient Specific Functional Scale; SD, standard deviation; VAS, Visual Analogue Scale.

^†^Lower values indicate lower follow-up scores adjusting for baseline values in the flexible belt group.

Combining the two groups, PSFS scores decreased from baseline (mean ± SD 6.8 ± 1.6) to follow-up (4.5 ± 2.7) with an overall mean decrease of 2.3 (95% CI 1.2 to 3.5, p < 0.001). Scores reduced by 2.3 (flexible) and 2.4 points (rigid), equating to a reduction of 36% and 34%, respectively. The activities that these women had particular difficulty with were rolling over in bed (n = 11/20), walking (for a variable time, n = 8/20), and getting up from sitting (after a variable period spent sitting, n = 7/20). Pain VAS scores (preceding week) also decreased overall (12.8 mm, 95% CI 2.5 to 23.0, p = 0.018). Pain VAS (preceding 24 hours) was not examined in this combined analysis due to the evidence for different effects between the groups described above. There was no significant change in overall MODQ scores when the two groups were combined (p = 0.243). However, at baseline, four individuals were classified as having minimal disability due to pregnancy-related symphyseal pain, 12 had moderate disability and four, severe disability; at week 3, six individuals had minimal disability, 11 moderate and three severe.

**Figure 3:**
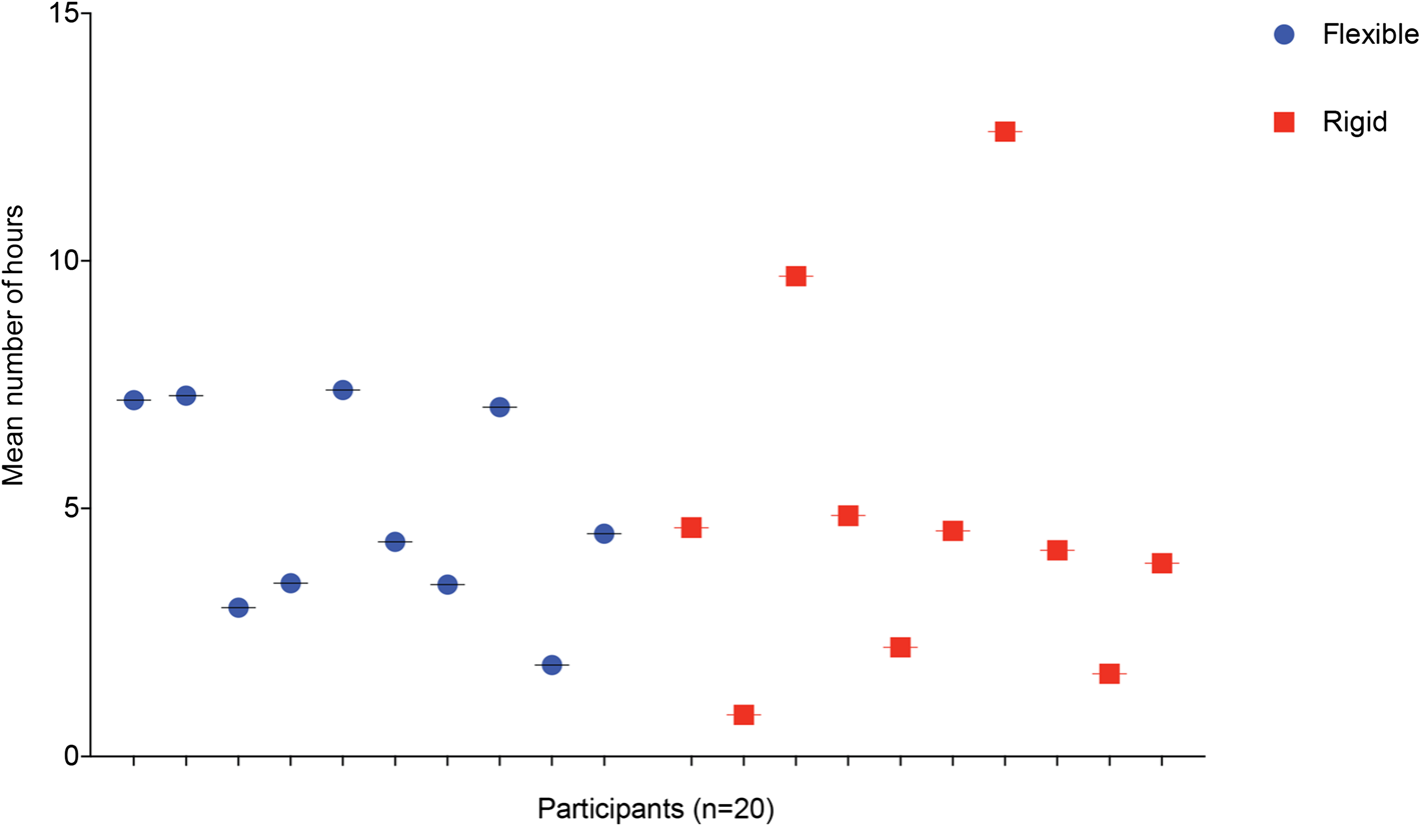
**Mean number of hours each participant wore the belt per day, over a 3-week period.** The mean duration of daily use ranged from 0.8 ± 1.4 to 12.6 ± 1.9 hours. These data are based on the information returned by each participant in response to daily text messages.

The mean ± SD number of times a participant responded to the daily text message over the 3-week period was 18.9 ± 4.1 (range 14 – 21; one outlier 3). Pelvic belts were worn for an average of 4.9 ± 2.9 hours per day but there was no significant difference in duration of belt use between the two groups (mean 5.0 ± 2.1 hours for flexible belt and 4.9 ± 3.6 hours for rigid belt, p = 0.973) (Table 2). Analyzing all participants, longer periods of belt use were associated with a greater decrease in pain VAS (preceding week) with a 3.9 mm greater decrease per additional hour worn (95% CI 0.8 to 6.9, p = 0.016) but no difference in PSFS (p = 0.546) or MODQ (p = 0.096). There was considerable variation in duration of belt usage between individuals, ranging between a mean of 12.6 ± 1.9 hours and 0.8 ± 1.4 hours (Figure 3). After wearing the alternate belt for a week, the flexible belt was deemed most comfortable by 14 of the 17 (82%) participants, with reports of the rigid belt tending to “ride up”, move out of position when sitting down, and being uncomfortable and “digging in” whilst sitting. No adverse events were reported.

## Discussion

The effect of pelvic support belts for pregnancy-related low back pain [4] and/or pelvic pain [5,6,10,21,24,26-28] and more specifically, pubic symphyseal pain (PSP) [14] has been previously investigated but little is known of comfort, and the frequency and length of time pregnant women are prepared to wear belts for. Our study provides novel pilot data indicating that the use of a pelvic belt may improve daily functional activity as assessed by the PSFS over a period of three weeks, and the use of a flexible belt could decrease pain (preceding 24 hours, VAS) more than a rigid belt (p = 0.049), but these findings remain to be confirmed in a larger randomized controlled trial with the inclusion of an appropriate control. On average, women with pregnancy-related PSP wore the belts for approximately 5 hours a day and found the wider, more flexible belt more comfortable than the rigid belt.

Combined data from both groups (flexible and rigid belt) showed an improvement in function (as assessed by the PSFS), and pain (as assessed by the VAS), supporting the carry-forward of pelvic belts for future investigation. This is similar to improvements documented in a previously published randomized controlled trial, observing the effect of a support garment on low back pain and posterior pelvic pain in pregnant women [3]. In the current study, PSFS scores decreased by 36% and 34% for the flexible and rigid belts, respectively, similar to the improvements documented by Depledge et al. [14] (rigid, 30%; flexible, 25%). One of the difficulties in assessing the efficacy or effectiveness of pelvic support belts relates to the fact that they are rarely used in isolation [8-10,14,22,24,26], resulting in contamination of the findings by the additional variables of exercise and advice [14,26]. In this study, all participants were given the same advice and there was no specific instruction to exercise. The flexible belt produced a mean 2.3-point difference and the rigid belt a mean 2.4-point difference over the intervention period. This change may be deemed to be clinically significant [31] however, it must be confirmed in a larger sample.

Our result showing a reduction in pain for the flexible pelvic belt intervention (preceding 24 hours, VAS) compared to the rigid belt in our preliminary sample is promising in terms of symptomatic management of pregnancy-related PSP. The magnitude of this reduction (40%) is likely to be clinically significant and for a woman with typical baseline scores for pain in the study (overall mean 56.9), this would represent a 0.9 standard deviation relative decrease in the flexible belt group compared to the rigid belt group, greater than the 0.8 standard deviations often regarded as a “large” effect. Furthermore, the women preferred the flexible type of belt to the rigid belt in terms of comfort. The mechanism of this benefit is uncertain. The use of ankle strapping has been shown to improve foot position awareness and may help to prevent ankle sprains in athletes [36]. The same proprioceptive mechanisms may be influencing behavior in individuals with PSP; the presence of an external device (in this case, a pelvic support belt) potentially makes individuals more aware of their activity and consequently modifies behavior to minimize or avoid pain.

Although differences within- or between-group mean values for MODQ were not statistically significant, the noted improvements may still be of clinical significance. Fourteen individuals improved their scores by week 3 and three were unchanged. Four individuals changed from “moderate” to “minimal disability” and two from “severe” to “moderate disability”. Further investigation of MODQ as an outcome measure for PSP interventions is therefore warranted in a larger study to obtain more precise estimates of its effect.

The duration that women wore the belts for was highly variable, and the average daily use of five hours was less than anticipated. Depledge et al., [14] recorded 6.5 and 6.1 hours a day for the rigid and flexible belts, respectively. A possible explanation of the low mean daily use may relate to the intermittent nature of PSP symptoms and a tendency for women to wear belts only if they had pain. Our data do not provide an insight into whether the belts were only utilized in response to pain or were worn as a prophylactic measure. We recommended that participants wore the pelvic support belt for as long as they were comfortable, but prescribed no specific duration since there are no existing guidelines on duration of belt use and efficacy. The high proportion of participants that responded to text-messaging suggests that this modality of communication may be a reliable method of self-monitoring [37,38] when compared to daily self-report diaries [8].

The flexible belt was the favored belt in terms of comfort (82%). In contrast, Depledge et al., [14] reported that only 27% of individuals who wore the rigid belt found it uncomfortable, compared to 43% of those who wore the flexible belt, suggesting that the rigid belt was better received. However, as participants in the study by Depledge et al. [14] trialed only one type of belt, they were unable to make an individual comparison. Other intervention studies have only explored the effects of a single type of pelvic support belt or garment [3,5,10,27]. The practicalities highlighted by the participants in the current study, such as being able to keep the belt in place when getting up from sitting and making sure that it is comfortable to wear while sitting, should be considered when choosing the most suitable belt for a larger investigation into their effect in pregnancy-related PSP symptoms.

The present study has some limitations. Given that this was a pilot study, interpretation of the data is somewhat limited by the small number of participants and the consequently wide confidence intervals. This restriction should be taken into account when interpreting results. Another limitation is the lack of a control group as it was intended only to directly compare flexible and rigid belts. Consequently, changes in mean PSFS scores (2.3 lower at follow-up) and mean pain VAS over the preceding week (12.8 mm lower at follow-up) need to be interpreted with caution. However, it is unlikely that such a decrease in PSFS (greater than the clinically meaningful change of 2 points suggested by Westaway et al. [31]) would have occurred in the absence of any intervention given the typical gestational age of the participants (mean 30.8 weeks). The design of our study preceded the recent publication of the Pelvic Girdle Questionnaire [13] and therefore this outcome measure could not be incorporated.

Based on these data we would recommend the PSFS as the primary outcome measure for a larger trial, with the VAS (preceding 24 hours) as a secondary outcome. A 2.3-point difference in PSFS observed in this study is a clinically important change [31]. Based on these pilot data, an adequately powered randomized controlled clinical trial, assuming correlations between baseline and follow-up values of 0.5 or higher (based on the observed correlation in the present study of 0.61), would require approximately 50 participants in each arm with full data to have 80% power to detect a moderate (0.5 SD, equivalent to around a 2.3 difference in PSFS scores based on standard deviations from the current study) difference in PSFS scores, which we consider to be clinically important, using a two-sided test at the 0.05 level. The results of this pilot study support a larger-scale study to determine the functional effectiveness of flexible pelvic belts as a treatment for PSP symptoms.

## Conclusion

The results of the current pilot study suggest that in general, pelvic support belts may have the potential to reduce pain and improve function in pregnant women with pubic symphyseal pain and that the flexible belt may be more effective and more comfortable. A larger study would be needed to definitively confirm these findings and to assess the potential benefits of pelvic support belts alongside other therapeutic interventions such as exercise regimens.

## Additional files

Additional file 1:
**Details of the three functional tests performed to confirm the presence of pubic symphysis pain description of data: descriptions of testing procedures [** 11 **,** 12 **,** 14 **,** 25 **].**
Additional file 2:
**Baseline questionnaire description of data: questionnaires used in the baseline assessment of participants.** Includes text and one figure. Additional file 3:
**Weekly phone interview follow-up questions description of data: questions used to assess participants at weeks 1, 2, 3 and 4 [** 28 **-** 31 **].**

## Abbreviations

MODQ: Modified oswestry disability questionnaire
PSFS: Patient specific functional scale
SD: Standard deviation
VAS: Visual analogue scale

## References

[1] Albert H, Godskesen M, Westergaard J (2001). Prognosis in four syndromes of pregnancy-related pelvic pain. Acta Obstet Gynecol Scand.

[2] Mens JM, Pool-Goudzwaard A, Stam HJ (2009). Mobility of the pelvic joints in pregnancy-related lumbopelvic pain: a systematic review. Obstet Gynecol Surv.

[3] Kalus SM, Kornman LH, Quinlivan JA (2008). Managing back pain in pregnancy using a support garment: a randomised trial. BJOG.

[4] Carr CA (2003). Use of a maternity support binder for relief of pregnancy-related back pain. J Obstet Gynecol Neonatal Nurs.

[5] Östgaard HC, Zetherström G, Roos-Hansson E, Svanberg B (1994). Reduction of back and posterior pelvic pain in pregnancy. Spine.

[6] Norèn L, Ostgaard S, Nielsen TF, Östgaard HC (1997). Reduction of sick leave for lumbar back and posterior pelvic pain in pregnancy. Spine.

[7] Ronchetti I, Vleeming A, van Wingerden JP (2008). Physical characteristics of women with severe pelvic girdle pain after pregnancy: a descriptive cohort study. Spine.

[8] Elden H, Ladfors L, Olsen MF, Ostgaard HC, Hagberg H (2005). Effects of acupuncture and stabilising exercises as adjunct to standard treatment in pregnant women with pelvic girdle pain: randomised single blind controlled trial. BMJ.

[9] Elden H, Fagevik-Olsen M, Ostgaard HC, Stener-Victorin R, Hagberg H (2008). Acupuncture as an adjunct to standard treatment for pelvic girdle pain in pregnant women: randomised double-blinded controlled trial comparing acupuncture with non-penetrating sham acupuncture. BJOG.

[10] Haugland KS, Rasmussen S, Daltveit AK (2006). Group intervention for women with pelvic girdle pain in pregnancy. A randomized controlled trial. Acta Obstet Gynaecol Scand.

[11] Albert H, Godskesen M, Westergaard J (2000). Evaluation of clinical tests used in classification procedures in pregnancy-related pelvic joint pain. Euro Spine J.

[12] Röst CC, Jacqueline J, Kaiser A, Verhagen AP, Koes BW (2004). Pelvic pain during pregnancy: a descriptive study of signs and symptoms of 870 patients in primary care. Spine.

[13] Stuge B, Garratt A, Jenssen HK, Grotle M (2011). The pelvic girdle questionnaire: a condition-specific instrument for assessing activity limitations and symptoms in people with pelvic girdle pain. Phys Ther.

[14] Depledge J, McNair PJ, Keal-Smith C, Williams M (2005). Management of symphysis pubis dysfunction during pregnancy using exercise and pelvic support belts. Phys Ther.

[15] Owens K, Pearson A, Mason G (2002). Symphysis pubis dysfunction–a cause of significant obstetric morbidity. Eur J Obstet Gynecol Reprod Biol.

[16] Mens JM, Vleeming A, Stoeckart R, Stam HJ, Snijders CJ (1996). Understanding peripartum pelvic pain. Implications of a patient survey. Spine.

[17] MacLennan AH, MacLennan SC (1997). The Norwegian Association for Women with Pelvic Girdle Relaxation (Landforeningen for Kvinner Med Bekkenlosningsplager). Symptom-giving pelvic girdle relaxation of pregnancy, postnatal pelvic joint syndrome and developmental dysplasia of the hip. Acta Obstet Gynaecol Scand.

[18] Vleeming A, Albert HB, Ostgaard HC, Sturesson B, Stuge B (2008). European guidelines for the diagnosis and treatment of pelvic girdle pain. Eur Spine J.

[19] Kanakaris NK, Roberts CS, Giannoudis PV (2011). Pregnancy-related pelvic girdle pain: an update. BMC Med.

[20] Vleeming A, Volkers ACW, Snijders CJ, Stoeckart R (1990). Relation between form and function in the sacroiliac joint. Part I: Clinical anatomical aspects. Spine.

[21] Snijders CJ, Vleeming A, Stoeckart R (1993). Transfer of lumbosacral load to iliac bones and legs. Part 1: Biomechanics of self-bracing of the sacro-iliac joints and its significance for treatment and exercise. Clin Biomech.

[22] Stuge B, Lærum E, Kirkesola G, Vøllestad N (2004). The efficacy of a treatment program focusing on specific stabilizing exercises for pelvic girdle pain after pregnancy: a randomized controlled trial. Spine.

[23] Vleeming A, Buyruk H, Stoeckart R, Karamursel S, Snijders CJ (1992). An integrated therapy for peripartum pelvic instability: a study of the biomechanical effects of pelvic belts. Am J Obstet Gynecol.

[24] Mens JM, Snijders CJ, Stam HJ (2000). Diagonal trunk muscle exercises in peripartum pelvic pain: a randomized clinical trial. Phys Ther.

[25] Mens JM, Vleeming A, Snijders CJ, Stam HJ, Ginai AZ (1999). The active straight leg raising test and mobility of the pelvic joints. Euro Spine J.

[26] Nilsson-Wikmar L, Holm K, Öijerstedt R, Harms-Ringdahl K (2005). Effect of three different physical therapy treatments on pain and activity in pregnant women with pelvic girdle pain: a randomized clinical trial with 3, 6, and 12 months follow-up postpartum. Spine.

[27] Mens JM, Damen L, Snijders CJ, Stam HJ (2006). The mechanical effect of a pelvic belt in patients with pregnancy-related pelvic pain. Clin Biomech.

[28] Price DD, McGrath PA, Rafii A, Buckingham B (1983). The validation of visual analogue scales as ratio scale measures for chronic and experimental pain. Pain.

[29] Fritz JM, Irrgang JJ (2001). A comparison of a modified oswestry Low back pain disability questionnaire and the quebec back pain disability scale. Phys Ther.

[30] Roland M, Fairbank J (2000). The roland-morris disability questionnaire and the oswestry disability questionnaire. Spine.

[31] Westaway MD, Stratford PW, Binkley JM (1998). The patient-specific functional scale: validation of its use in persons with neck dysfunction. J Orthop Sports Phys Ther.

[32] Juul-Kristensen B, Røgind H, Jensen DV, Remvig L (2007). Inter-examiner reproducibility of tests and criteria for generalized joint hypermobility and benign joint hypermobility syndrome. Rheumatology.

[33] Hakim A, Grahame R (2003). Joint hypermobility. Best Pract Res Clin Rheumatol.

[34] Damen L, Spoor CW, Snijders CJ, Stam HJ (2002). Does a pelvic belt influence sacroiliac joint laxity?. Clin Biomech.

[35] Fairbank JC, Couper J, Davies JB, O’Brien JP (1980). The Oswestry low back pain disability questionnaire. Physiotherapy.

[36] Robbins S, Waked E, Rappel R (1995). Ankle taping improves proprioception before and after exercise in young men. Br J Sports Med.

[37] Shapiro JR, Bauer S, Andrews E, Pisetsky E, Bulik-Sullican B, Hamer RM (2010). Mobile therapy: Use of text-messaging in the treatment of bulimia nervosa. In J Eat Disord.

[38] Haug S, Meyer C, Schorr G, Bauer S, John U (2009). Continuous individual support of smoking cessation using text messaging: a pilot experimental study. Nicotine Tobo Res.

