# Supplementary material for: Adherence, tolerance and effectiveness of two different pelvic support belts as a treatment for pregnancy-related symphyseal pain - a pilot randomized trial
Source: BMC Pregnancy Childbirth. 2015 Feb 15;15:36. doi: 10.1186/s12884-015-0468-5 (PMC4339641; doi:10.1186/s12884-015-0468-5)
Supplement: Additional file 1: — Details of the three functional tests performed to confirm the presence of pubic symphysis pain description of data: descriptions of testing procedures [ 11 , 12 , 14 , 25 ]. [file 12884_2015_468_MOESM1_ESM.docx]

Additional File 1. Details of the three functional tests performed to confirm the presence of pubic symphysis pain.

1. Reproduction of pain from palpation.

Tested in supine and performed as has been described previously [11] . The duration of pain was used to determine whether the patient experienced pain (i.e. lasting for more than 5 seconds) or tenderness (i.e. lasting for less than 5 seconds).

2. Modified Trendelenburg’s Test.

Tested in standing and performed as described previously [11]. Participants reported on the presence of pain in the PS and were assessed for the presence of contralateral hip drop.

3. Active Straight Leg Raise Test.

Tested in supine and performed as described previously [14, 12, 25]. Participants reported on the presence of pain in the pubic symphysis and their ability to perform the task using a Likert Scale (0, able to perform without difficulty; 5, able to perform with extreme difficulty).
