# Supplementary material for: Adherence, tolerance and effectiveness of two different pelvic support belts as a treatment for pregnancy-related symphyseal pain - a pilot randomized trial
Source: BMC Pregnancy Childbirth. 2015 Feb 15;15:36. doi: 10.1186/s12884-015-0468-5 (PMC4339641; doi:10.1186/s12884-015-0468-5)

Additional File 2: Baseline Questionnaire

Pelvic Belt Study Questionnaire: Pain in the joint at the front of the pelvis during pregnancy

Surname Given Names

Address

Midwife/GP Name

How can we best contact you?

Home phone Time: am pm

Mobile phone Time: am pm

Work phone Time: am pm

Date of Birth

Height Weight (just before pregnancy)

How many children do you have?

What is the date you are due to have/had your baby?

Which ethnic group do you belong to? (please tick one or more of the following boxes:)

NZ European

Maori

Samoan

Cook Island Maori

Tongan

Niuean

Chinese

Indian

Other (please specify)

What is your current employment status?

Full-time paid employment/ Full-time self employment

Part-time employment (less than 40 hours/week)

Maternity leave

Homemaker

Other (please specify)

What amount of physical activity do you undertake per week (30 minutes or more/day)?

None

Once or twice a week

Three or four times a week

More than four times a week

What sort/type of exercise do you partake in?

Do you currently experience localized pain in the joint at the front of your pelvis (pubic symphysis) during routine daily activities, at least three times per week?

Yes

No

If yes, how long have you had this pain for?

Less than a week

1 to 3 weeks

3 to 6 weeks

6 weeks to 3 months

More than 3 months

Do you also experience pain at the back of your pelvis or in your lower back, and if so, is the pain at the front of your pelvis the worst?

Yes

No

Have you suffered from localized pain in the joint at the front of your pelvis (pubic symphysis) previously?

Yes

No

If yes, was this when you were pregnant? (please specify)

Have you suffered from low back pain previously?

Yes

No

Do you currently have low back pain?

Yes

No

Please mark an X on the line below to represent your worst pubic symphysis pain (pain in the joint at the front of your pelvis) experienced during the last 24 hour period.

No pain Worst possible pain

Please mark an X on the line below to represent your worst pubic symphysis pain (pain in the joint at the front of your pelvis) experienced during the last week.

No pain Worst possible pain

Over a 24 hour period, how long would you estimate you have your pain for?

hours minutes

Please indicate on the following diagram, by shading or marking in the area(s), where you experience muscle and/or joint pain.


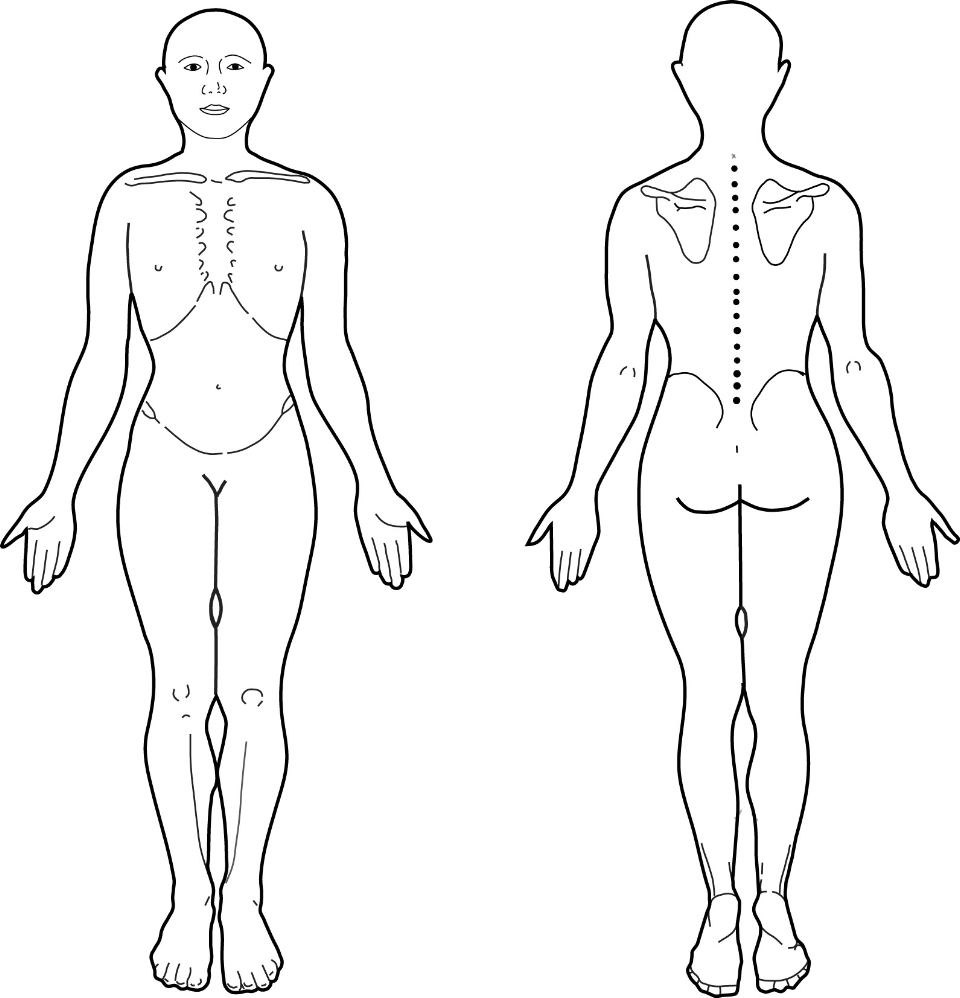

Supplement: Additional file 2: — Baseline questionnaire description of data: questionnaires used in the baseline assessment of participants. Includes text and one figure. [file 12884_2015_468_MOESM2_ESM.docx]
