# Supplementary material for: Adherence, tolerance and effectiveness of two different pelvic support belts as a treatment for pregnancy-related symphyseal pain - a pilot randomized trial
Source: BMC Pregnancy Childbirth. 2015 Feb 15;15:36. doi: 10.1186/s12884-015-0468-5 (PMC4339641; doi:10.1186/s12884-015-0468-5)
Supplement: Additional file 3: — Weekly phone interview follow-up questions description of data: questions used to assess participants at weeks 1, 2, 3 and 4 [ 28 - 31 ]. [file 12884_2015_468_MOESM3_ESM.docx]

**Additional File 3 – Weekly phone interview follow-up questions.**

1. Can you tell me approximately how many hours per day you used the belt in the past week?

2. Have you found the belt comfortable to wear? (yes/no/sometimes and comments)

3. Is the belt easy to use (e.g. get on/off)? (yes/no/sometimes and comments)

4. Has wearing the belt reduced your pain at all? (yes/no/sometimes and comments)

5. Has wearing the belt made it easier to do any daily functional activities? (yes/no/sometimes and comments)

6. Patient Specific Functional Scale [31]

When I assessed you on (_____________)(date of previous visit), you told me that you had difficulty with 1. , 2. , 3. . (read activities identified at baseline assessment). Today, do you still have difficulty with each of these activities?

On a scale of 0 to 10, with 0 being unable to perform the activity and 10, able to perform the activity at a pre-pregnancy level, please rate your ability in performing each of the activities you previously identified.

| **Activity** | **Follow-up Rating (0-10)** |
| --- | --- |
| 1. |  |
| 2. |  |
| 3. |  |
| Additional |  |
| Additional |  |

**Additional phone interview follow-up questions – Week 3**

1. If you had a choice, would you continue to wear this belt? (yes/no/sometimes and comments)

2. With regard to overall effectiveness (either reducing your pain or meaning you are better able to do normal daily activities), please score the belt on the following 5-point scale:

1. Very effective

2. Effective

3. Somewhat effective

4. Not effective

5. Very not effective

Participants also re-completed a MODQ [29, 30] and VAS scales [28] for the past week and previous 24 hours.

**Additional phone interview follow-up questions – Week 4**

9. Which of the two belts that you trialled was most comfortable? (belt 1/ belt 2/ no difference and comments)

10. Which of the two belts was the easiest to use? (belt 1/ belt 2/ no difference and comments)

11. Which of the two belts was best in reducing your pain or making it easier to do normal daily activities? (belt 1/ belt 2/ no difference and comments)
